# South London Practitioners and the Removal of King's College Hospital

**Published:** 1908-05-09

**Authors:** 


### HOSPITAL ADMINISTRATION.

### CONSTRUCTION AND ECONOMICS.

### SOUTH LONDON PRACTITIONERS AND THE REMOVAL OF KING'S COLLEGE HOSPITAL.

In connection with the removal of King's College Hospital to Denmark Hill, a largely attended meeting of medical practitioners was held in Peckham Town Hall on Thursday, April 30, Dr. Robert Capes, Chairman of the South London Hospitals Committee, presiding.

In opening the proceedings the Chairman said they were met to discuss the question of the admission of paying patients into the wards of general hospitals, and also a scheme for limiting the number of out-patients by a system of certificates signed by local practitioners. The South London Hospitals Committee was elected in 1903 by the Lambeth, Norwood, and Wandsworth divisions of the British Medical Association, the Brixton Medical Society, the Camberwell Provident Dispensary, and the South-West London Medical Society. The Committee had nothing to do with opposing the coming to Camberwell of King's College Hospital—that was an established fact; but it had discussed questions affecting the hospital and the medical profession of the locality with the Watching Committee of King's College Hospital, a sub-committee representing the hospital, but without executive powers, appointed by the Board of the Medical School and acting as a go-between to adjust arrangements between the hospital and the local practitioners. Its procedure was to pass on recommendations to the various hospital committees. Their relations had been most cordial and friendly; they had held numerous conferences, and eventually the South London Committee had drawn up seventeen resolutions, of which fifteen had been accepted by the Watching Committee. The first five were as follows:—

(1) That there be no issue of subscribers' letters; (2) that sickness and poverty be the qualifications for hospital treatment; (3) that no charge be made for the treatment of any patient of the hospital, or for the supply of medicine or dressings; (4) that urgent cases receive attention without inquiry; (5) that each applicant for treatment, on the first visit, be seen by a registered medical practitioner, and, if found suitable for hospital treatment, attended to; and that the name, age, address, occupation, or position be entered in a register.

So far they were in agreement, but the Watching Committee did not approve of the next: (6) That, on a second application for treatment a patient bring with him a recommendation signed by a qualified medical practitioner, stating that such patient is a suitable person for hospital treatment.

Subject to some arrangement in that matter, the two committees were in agreement as to the next ten resolutions. (7) That such certificate be written on a form supplied by the hospital; (8) That such certificate, duly filled up, be appended to each patient's prescription-paper or attendance-card; (9) that the following be submitted to the authorities of King's College Hospital as the ordinary form to be supplied to patients attending the hospital for the first time: "Medical Certificate: I hereby recommend............ aged.........as a suitable case for treatment at the................ Hospital. ....................................Registered Practitioner. ..................19............ No charge is made for this certificate." Reverse Side of Certificate: "In signing this certificate the practitioner is requested to keep in view two points: (*i*) That the applicant is not in a position to pay for treatment; (*ii*) That the case is, from a hospital point of view, suitable for treatment." (10) That a similar certificate be required in maternity cases; (11) that it would be satisfactory if a system could be arranged whereby obscure and interesting cases might be brought or sent with a note by practitioners, for an opinion when a consultation fee cannot be paid; (12) that all cases of trivial accident or illness deemed ineligible for the out-patient department shall, after having been once attended to, be referred for treatment elsewhere; (13) that, should a general practitioner report in writing the attendance of a person well able to pay, the letter, if duly signed, be treated as confidential and the matter investigated; (14) that the hospital authorities use every means in their power to prevent unsuitable cases receiving hospital treatment, and appoint such officers as they consider desirable to make inquiries with a view to checking hospital abuse; (15) that the regulations as to the admission of patients for treatment be displayed in conspicuous positions in the hospital; (16) that no patients except maternity cases be attended outside the hospital.

The Watching Committee did not approve the final resolution: (17) That private wards or nursing homes for paying patients be distinct from the general hospital, and not erected or maintained out of the funds given for hospital purposes.

Having thus stated the position of affairs, the Chairman called on Dr. Biggs, who moved:

> That this meeting strongly disappoves of paying patients being admitted into general teaching hospitals erected out of charitable funds, and sincerely hopes the authorities of King's College Hospital will not adopt any system for the admission of paying patients.

He emphasised the friendly character of the relations between the two committees, and thought the general practitioners of the neighbourhood owed a debt of gratitude to the King's College Hospital for coming to the practitioners, taking their opinions and endeavouring to adjust differences between them. He believed such an attitude was unprecedented, and he was certain that nothing that would be said or done at that meeting would be antagonistic to the Watching Committee or the hospital. However, as he understood it, the idea of the hospital was that while they did not propose to take those who could afford to pay for their keep and for the cost of treatment, they were prepared to take another class, better in position than the ordinary hospital patient, whom they called contributory patients. These were to be accommodated separately, and it was to be left to the staff whether they would attend them or not. The objection to this, he held, was that the funds supplied by the public for the treatment of the sick poor should not be applied to the commercial purpose of obtaining funds for the hospital, as was being done at St. Thomas's and at Guy's. He thought all such cases should be treated outside in nursing homes or cottage hospitals, and it had been suggested to him by a well-known consultant that medical men should combine and manage these separate institutions without outside or lay influence at all. He was of opinion that the younger men, while waiting for their practice to grow, would come down to such places for very moderate fees.

Dr. Silk, the Secretary of the King's Watching Committee, said it would be fully three or four years before there would be any prospect of getting patients into the new hospital, so that there was really no urgency in the matter. In his opinion the real position was that the laity all over the country were urging the hospitals to take paying patients, and he thought the public had a right to say that those who paid the piper should call the tune. They had, however, taken care to limit the space available for contributing patients, and he thought there was no reasonable probability of the contributing element interfering with the interests of the general practitioners in the neighbourhood, the proportion being kept small. As to the amount to be paid per week, nothing had yet been fixed; but it was to be merely rent, and not to be made a matter of gain to the hospital; it would certainly be something small, certainly not five or ten or twenty guineas.

Dr. Barker Smith wanted the fact recognised that the public was taking a sane view in wishing to send special cases to hospitals. It was rarely that people had the convenience or could afford to deal with them at home, nor could the practitioner give the best possible talent or had the apparatus pertaining to the study or treatment of such cases.

Dr. Key said they had heard that the people who supplied the money should rule in this matter, but he thought there were higher considerations, and that they ought not to give way to uninstructed opinion merely because it had money behind it.

Dr. Biggs (the mover), in replying on the discussion, quite agreed with the last speaker. The public was always pressing the medical profession to do something for nothing, merely on the ground that it was "a noble profession."

The resolution was agreed to.

Dr. Esler moved:

That it is the opinion of this meeting that the system of admission to King's College Hospital by certificates from the medical practitioners, as set forth in resolution six of the South London Hospitals Committee, should be given a fair trial, and this meeting strongly urges the authorities of King's College Hospital to incorporate this system into the rules of the hospital.

The position was that hospitals generally wanted more funds and had too many out-patients, and if this plan were adopted, there would be an improvement in both respects. It was time medical practitioners moved in their own interests. By means of the British Medical Association much had been done to safeguard the medical profession, but much remained before they could claim that in all matters medical "Hippocrates is King."

Dr. E. Rowland Fothergill moved as an amendment:

1. That this meeting of South London registered medical practitioners, understanding (1) that at the present time a Royal Commission is inquiring into the Poor-law system; (2) that the King Edward Fund has decided to inquire into the system prevailing at the large general hospitals of London with regard to the admission of out-patients; and (3) that for the uses of the sick poor resident in Camberwell and district there are already provided twenty-six hospitals, five infirmaries, eighteen dispensaries, and nine other institutions; considers that there has been no evidence offered of the necessity for any out-patient department at the proposed King's College Hospital on the lines of those at the large London general hospitals, regrets that a contract for £65,000 for such a department has been placed; and is of opinion that a small out-patient department to which will be admitted either for consultation or treatment only those recommended as suitable by registered medical practitioners as suggested in resolutions 6, 7, 8, 9, and 11, would amply meet the needs of the district, would fulfil all requirements for the education of students, and should be given a fair trial.
2. That the Lambeth, Norwood, and Wandsworth Divisions, the Metropolitan and South-Eastern Branch Councils, as well as the Central Council and Representative Body of the Association, be urged to approve and support this resolution, a copy of which shall also be sent to King Edward Hospital Fund, the Hospital Saturday and Sunday Funds, the Charity Organisation Society, King's College Hospital Board, and the Press.

He held it to be a fundamental rule that the interest of the profession was the interest of the public; that any scheme which pauperised the public would pauperise the profession, and that any scheme which would lift the profession would lift the public also. He pointed out the great increase that had taken place in the number of out-patients in consequence of special appeals, and declared that touting for money meant touting for out-patients. They in South London did not want another out-patient department, but they wanted collaboration between King's and the existing institutions. He believed if the out-patient department were turned into a consulting department it would be much more useful to the students.

Dr. Cocks seconded the amendment.

Dr. McManus strongly recommended the system in practice at the Bolingbroke Hospital, where the committee had done its best to meet the wishes of the general practitioners, of whom there were seven on the Board of Governors. They had some well-known consultant for each department. The general practitioner gave the patient a card briefly describing his case; the consultant in charge of the department examined him and sent him to the practitioner for treatment. So that he got a first-class opinion for nothing, while the consultant got a set of specially selected interesting and doubtful cases. Accidents, of course, were treated at once, but if they could afford it they were given a card for a medical man, to be dressed in future by him. They found that in practice that system worked well, and if good for the Bolingbroke, how much better it would be for the great general hospitals. If the consultant wrote his diagnosis and indicated the treatment, the patient would be far better and more honestly looked after.

Dr. Hogarth thought there were difficulties in the way of asking the medical man to sign the certificate, and suggested the almoner should write to him, and he should reply by post.

Dr. Harvey Norton said that at the Belgrave Hospital they had tried the certificate system for some months, but it had failed utterly. He suggested that the medical men should subscribe, and themselves keep an almoner at each hospital.

Dr. Key thought the abuse complained of was largely due to contract men sending patients to hospitals in large numbers; if they had to write a brief diagnosis they would take more care. The difficulty of dealing with the sick poor must be met by a grant from the State.

Dr. Partridge thought that medical practitioners should be supplied with books of certificates to give to suitable patients.

Dr. Fothergill considered the profession generally asked too big fees. Often if a moderate fee were asked the patient would not want to go to the hospital at all.

The amendment was put to the meeting and carried by an overwhelming majority; on being put as a substantive motion it was adopted *nem. con.*

Dr. Soper moved a resolution approving the action of the South London Hospitals Committee, and urging that a deputation should approach King's College Hospital in the matter. This also was agreed to, and the customary votes of thanks concluded the proceedings.

